# A Novel Mechanism Underlying the Inhibitory Effects of Trastuzumab on the Growth of HER2-Positive Breast Cancer Cells

**DOI:** 10.3390/cells11244093

**Published:** 2022-12-16

**Authors:** Hamid Maadi, Zhixiang Wang

**Affiliations:** Signal Transduction Research Group, Department of Medical Genetics, Faculty of Medicine and Dentistry, University of Alberta, Edmonton, AB T6G 2H7, Canada

**Keywords:** EGF, EGFR, HER2, trastuzumab, dimerization, phosphorylation, lipid raft, akt, erk, cell proliferation, cell cycle progression

## Abstract

To improve the efficacy of trastuzumab, it is essential to understand its mechanism of action. One of the significant issues that makes it difficult to determine the precise mechanism of trastuzumab action is the formation of various HER receptor dimers in HER2-positive breast cancer cells. So far, studies have focused on the role of HER2–HER3 heterodimers, and little is known regarding EGFR–HER2 heterodimers. Here, we study the role of trastuzumab on the cell signaling and cell proliferation mediated by EGFR–HER2 heterodimers in BT474 and SRBR3 cells. EGF stimulates the formation of both EGFR homodimer and EGFR–HER2 heterodimer. Trastuzumab only binds to HER2, not EGFR. Therefore, any effects of trastuzumab on EGF-induced activation of EGFR, HER2, and downstream signaling proteins, as well as cell proliferation, are through its effects on EGFR–HER2 heterodimers. We show that trastuzumab inhibits EGF-induced cell proliferation and cell cycle progression in BT474 and SKBR3 cells. Interestingly trastuzumab strongly inhibits EGF-induced Akt phosphorylation and slightly inhibits EGF-induced Erk activation, in both BT474 and SKBR3 cells. These data suggest the presence of a novel mechanism that allows trastuzumab to inhibit EGR-induced Akt activation and cell proliferation, without blocking EGF-induced EGFR–HER2 heterodimerization and activation. We show that trastuzumab inhibits EGF-induced lipid raft localization of the EGFR–HER2 heterodimer. Disruption of the lipid raft with MβCD blocks HER2-mediated AKT activation in a similar way to trastuzumab. MβCD and trastuzumab synergically inhibit AKT activation. We conclude that trastuzumab inhibits EGF-induced lipid raft localization of EGFR–HER2 heterodimer, which leads to the inhibition of Akt phosphorylation and cell proliferation, without blocking the formation and phosphorylation of the EGFR–HER2 heterodimer.

## 1. Introduction

The human epidermal growth factor (EGF) receptor (EGFR) (HER) family (also known as ERBB Receptor family) is composed of four members including EGFR/HER1/ERBB1, HER2/ERBB2, HER3/ERBB3, and HER4/ERBB4 [1,2]. HER receptors are activated after homo- or hetero-dimerization [2,3,4]. While EGFR and HER4 are fully functional receptors, HER2 is unable to bind to a ligand and HER3 is kinase-dead. However, all HER receptors can be activated through heterodimerization among them [2,5,6,7,8,9]. HER2 is the only HER receptor that maintains a constitutively activated conformation in the absence of a ligand, which allows HER2 to form homodimers when overexpressed in cells. HER2 is also a preferred dimerization partner of other HER receptors [1,10]. The activated HER receptors, via multiple phosphotyrosine residues in the C-terminus, form complexes with signaling proteins, which stimulate various signaling pathways including Ras/ERK, PI3K/Akt, PLC-γ1, and STAT. By regulating these signaling pathways, HER receptors govern fundamental cellular processes [2,8,9,11].

The plasma membrane of the cell is not homogeneous. Certain types of lipids are distributed heterogeneously, which allows the selective enrichment or exclusion of distinct types of proteins, and thus leads to the formation of different compartmentalization structures. Lipid rafts are membrane microdomains enriched with cholesterol and sphingolipid, which makes them more rigid and tightly packed portions of the membrane [12]. Lipid rafts are also defined as the membrane domains that are resistant to solubilization by detergents such as Triton X-100 [13]. In cancer cells, lipid rafts act as signaling platforms by sequestering and concentrating oncogenic signaling components, which leads to cancer cell survival and invasion [14]. It is well established that EGFR and HER2 are localized to lipid rafts. While some research suggest that localization to a lipid raft negatively regulates EGFR signaling [15,16,17,18], most studies indicate that lipid rafts act as signaling hubs to positively regulate the activation of EGFR and HER2, and the activation of downstream signaling proteins including PI3K/AKT and Ras/ERK [19,20,21,22,23,24,25,26,27,28,29,30,31,32]. 

Overexpression of HER2 occurs in 20–30% of breast cancers [7,10,33,34,35,36,37]. Various therapies have been developed to target HER2 for treating HER2-positive breast cancers [38,39,40,41]. As the first HER2-targeted therapy approved by the FDA, trastuzumab selectively exerts antitumor effects in HER2-positive metastatic breast cancer patients [40,41]. Trastuzumab is a humanized recombinant monoclonal antibody to HER2, and selectively exerts antitumor effects in HER2-positive BC patients [40,41]. Trastuzumab binds to HER2 domain IV, close to the HER2 juxta-membrane region. While many mechanisms have been proposed for the antitumor activity of trastuzumab, including both extracellular and intracellular actions, the exact mechanisms are not known and may be context-dependent [9,40,41,42]. The extracellular action is through antibody-dependent cellular cytotoxicity (ADCC), which is well supported by numerous studies [43,44,45,46,47,48,49,50,51]. On the other hand, the data regarding the intracellular mechanisms have been controversial [9]. The two mostly studied intracellular mechanisms include: (1) inhibition of intracellular signal transduction leading to cell proliferation; and (2) inhibition of the proteolytic cleavage of HER2 ECD that produces oncogenic p95HER2 as discussed above [52,53,54,55,56]. While the first mechanism is the basis for developing trastuzumab, and has been referred by most reviews [40,57,58], it is not supported by experimental data, including our own [40,41,42,59,60,61,62,63,64].

While trastuzumab is the most used HER2 targeting agent, which has significantly improved outcomes, the overall response rate is low. Primary resistance is observed in >50% of HER-2+ patients treated with trastuzumab alone. For patients who initially responded to trastuzumab, the majority eventually develop acquired resistance [65,66]. To develop novel therapies to boost trastuzumab efficacy, it is critical to identify the mechanisms underlying trastuzumab action. 

One of the significant issues that makes it difficult to determine the precise mechanism of trastuzumab action is the formation of various HER receptor dimers in HER2-positive breast cancer cells. Previous studies regarding trastuzumab action and resistance in breast cancer have been focused on its role in the Akt pathway, in the context of HRG-induced HER2–HER3 heterodimers [8,60,67,68,69,70,71,72,73,74,75,76,77]. Recently, we studied the mechanism of trastuzumab in terms of HER2 homodimer. We showed that HER2 massively forms homodimers in CHO cells, stably expressing HER2 at high level. The formation of HER2 homodimer is not inhibited by the treatment of trastuzumab. We also showed that trastuzumab did not block the activation of HER2, as measured by the phosphorylation level of all the major HER2 tyrosine residues in the C-terminus [51,78]. However, little is known about the effects of trastuzumab on the signaling pathway mediated by EGF-induced EGFR–HER2 heterodimers.

Here we used two HER2-positive breast cancer cell lines, BT474 and SKBR3, to further understand the molecular mechanism underlying trastuzumab action in terms of EGFR and HER2 heterodimers. Both BT474 and SKBR3 cells overexpress EGFR, HER2 and HER3. EGF only binds to EGFR and stimulates the formation of both EGFR homodimer and EGFR–HER2 heterodimer. Trastuzumab only binds to HER2, not EGFR. Therefore, any effects of trastuzumab on EGF-induced activation of EGFR, HER2, and downstream signaling proteins, as well as cell proliferation, are through its effects on EGFR–HER2 heterodimers. We show that trastuzumab inhibits EGF-induced cell proliferation, and cell cycle progression in BT474 and SKBR3 cells. Interestingly trastuzumab strongly inhibits EGF-induced Akt phosphorylation, and slightly inhibits EGF-induced Erk activation in both BT474 and SKBR3 cells. These data suggest the presence of a novel mechanism that allows trastuzumab to inhibit EGR-induced Akt activation and cell proliferation, without blocking EGF-induced EGFR–HER2 heterodimerization and activation. We show that trastuzumab inhibits EGF-induced lipid raft localization of EGFR–HER2 heterodimer. Disruption of the lipid raft with MβCD blocks HER2-mediated AKT activation in a similar way to trastuzumab. MβCD and trastuzumab synergically inhibit AKT activation.

## 2. Materials and Methods

### 2.1. Cell Culture and Treatments

The cell lines used in this study include BT474 and SKBR3 cells purchased from ATCC (Manassas, VA, USA). All cells were grown in Dulbecco’s modified Eagle’s medium supplemented with 10% FBS and non-essential amino acids. Cells were maintained in a 5% CO_2_ atmosphere at 37 °C. C For the various treatments, the cells were serum-starved overnight and then exposed to EGF, trastuzumab, normal human IgG, CP-714724, Alex Fluor 488 conjugated cholera-toxin B-subunit (CT-B), and MβCD at indicated concentrations for indicated time periods. 

### 2.2. Chemicals and Antibodies

HER2 inhibitor CP-724714 was from Selleckchem (Houston, TX, USA). Normal human IgG were purchased from Sigma-Aldrich (St. Louis, MO, USA). Trastuzumab was from Roche (Basel, Switzerland). Mouse monoclonal antibodies to human EGFR (1005), HER2 (A-2) (sc-393712), human EGFR (1005), Akt1/2 (H-136), Erk1/2 (k-23), pT202/Y204 Erk1/2 were from Santa Cruz Biotechnology Inc. (Dallas, TX, USA). Rabbit monoclonal antibodies to phospho-Akt (Ser473) and phosphor(Thr308), and rabbit monoclonal antibody to HER2/ErbB2 (29D8) were purchased from Cell Signaling Technology (Danvers, MA, USA). Rabbit polyclonal antibodies to phospho-HER2 Y-1005, Y-1112, Y-1127, Y-1139, Y-1196, and Y-1248 were from FroggaBio (Toronto, ON, Canada). Secondary antibodies used for Western analysis, including Anti-rabbit and anti-mouse RDye^®^ 800 CW and RDye^®^ 650, were from LI-COR biotechnology Inc. (Lincoln, NE, USA). Invitrogen™ Cholera Toxin Subunit B (Recombinant) and Alexa Fluor™ 488 Conjugate were purchased from Fisher Scientific (Ottawa, ON, Canada). All other chemicals were purchased from Sigma-Aldrich (Oakville ON, Canada).

### 2.3. Cell Proliferation (Viability) Assay by MTT

The same number of cells (10^4^) were seeded in each well containing 200 μL DMEM with 10% FBS in a 96-well plate. After culture overnight, the cells were treated with EGF and/or various agents in fresh DMEM. Cell proliferation was assessed by MTT (3-(4,5-Dimethylthiazol-2-Yl)-2,5-Diphenyltetrazolium Bromide) assay using Vybrant MTT Cell Proliferation Assay Kit from Invitrogen (Grand Island, NY, USA) according to manufacturer’s instruction. The cell numbers were reflected by the color intensity at 540 nm wavelength which was measured by a microplate reader. 

### 2.4. Cell Cycle Analysis by Flow Cytometry

Cells were seeded in 6 cm plates in DMEM medium containing 10% FBS. After overnight culture, the cells were treated in fresh DMEM for 72 h, and then cell cycle was analyzed by flow cytometry. Briefly, the harvested cells were fixed in cold methanol for 20 min. The fixed cells were treated with 0.1% (*v*/*v*) Triton X-100 and 100 µg/mL RNase A in 1 mL PBS. The cells were centrifuged and resuspended in 1 mL PBS containing 0.1% (*v*/*v*) Triton X-100 and 15 µg/mL propidium iodide (PI) staining solution. The samples were kept in the dark for 30 min and DNA contents were measured with BD FACSCanto™ II. Finally, the results were analyzed with FlowJo V10 software (FlowJo, LLC, Ashland, OR, USA)

### 2.5. Cell Lysates and Immunoblotting

Cell lysates were prepared as previously described [51]. Briefly, the cells were collected by scraping and lysedin ice-cold Mammalian Protein Extraction Reagent (Pierce, Rockford, IL, USA) containing a protease and phosphatase inhibitor cocktail 0.02% NaN3, 0.1 mM 4-(2-aminoethyl)-benzenesulfonyl fluoride, 1 µM pepstatin A, 10 µg/mL aprotinin, 0.5 mM Na_3_VO_4_,). Following the incubation on ice for 1 h, the cell lysates were centrifuged at 21,000× *g* for 15 min at 4 °C. The supernatant was collected, mixed with equal volume of 2× sample buffer, and boiled for 5 min. Following the gel electrophoresis, the proteins were transferred to the nitrocellulose membrane. The nitrocellulose membrane was immunoblotted with various primary antibodies as indicated. The protein bands were detected and analyzed by using Odyssey CLx imaging system (LI-COR biotechnology Inc., Lincoln, NE, USA).

### 2.6. Immunofluorescence

Cells (10^5^) were seeded on 15 mm round cover glass in 24 well-plates, and were cultured for 48 h to allow the cells to settle and attach. Cells were treated in the same way as described in 5.1. Following the treatment, cells were washed with ice-cold PBS and then fixed with −20 °C methanol for 5 min. Afterwards, the cells were washed with TBS and blocked with TBS containing 1% bovine serum albumin (BSA) for 60 min. Cell were incubated in 2 µg/mL of primary antibodies, as indicated, for 60 min. The cells were washed and then incubated in 1 µg/mL FITC-conjugated and/or 1 µg/mL rhodamine-conjugated secondary antibodies for 60 min in the dark. Afterwards, the cells were washed with TBS and then incubated in 1 µg/mL DAPI solution for 5 min. The coverslips were mounted on microscope slides and were examined with a Deconvolution Microscope system (GE Healthcare Life Science, Saskatoon, SK, Canada). All of the images were deconvolved. The selected images were line scanned with the software SoftWoRx embedded in the Microscope system. The line scan measured the intrinsic intensity of the individual fluorescence channel, and was not affected by changing the contrast and brightness. 

### 2.7. Immunoprecipitation

Immunoprecipitation (IP) buffer [20 mM Tris-HCl pH 7.5, 150 mM NaCl, 1% NP-40, 0.1% sodium deoxycholate, 5 mM MgCl2, 0.5 mM Na3VO4, 100 mM NaF, 0.02% NaN3, 1 mM pepstatin A, 10 mg/mL aprotinin, and 0.1 mM 4-(2-aminoethyl)-benzenesulfonyl fluoride], were used to lysis the cells. The non-soluble cell debris was removed from cell lysates by centrifugation. Total proteins of 1 mg were incubated with 1 μg of primary antibody at 4 °C. After overnight incubation, the protein A conjugated with agarose was added to samples and incubated for 2 h at 4 °C. The immunoprecipitates were subjected to immunoblotting as described above.

### 2.8. Lipid Rafts Associated Proteins Isolation

The proteins present in lipid rafts were isolated from BT474 cells by Bio-Rad ReadyPrep™ Protein Extraction Kit (Signal) kit according to the instruction provided by manufacturer. Due to the presence of a high amount of cholesterol and sphingolipids in the lipid rafts, these regions of the cell membrane are insoluble in nonionic detergents. Therefore, treating the cells with nonionic detergents, provided by the kit, will solubilize hydrophilic regions of the cells, and leave the lipid raft microdomains and their associated proteins insoluble. This insoluble fraction can be solubilized in a specific buffer named protein solubilization buffer (PSB) provided by the company. 

### 2.9. Statistical Analysis

Blot band intensity was quantified by ImageJ software and normalized to total expression of protein of interest. Data were statistically analyzed by one-way analysis of variance (ANOVA) using Prism software (GraphPad Software, La Jolla, CA, USA). Data were presented as mean and standard deviation. *p* < 0.05 was considered as statistically significant.

## 3. Results

### 3.1. The Effect of Trastuzumab on the Proliferation and Cell Cycle Progression of HER2-Positive Breast Cancer Cells

**Figure 1:**
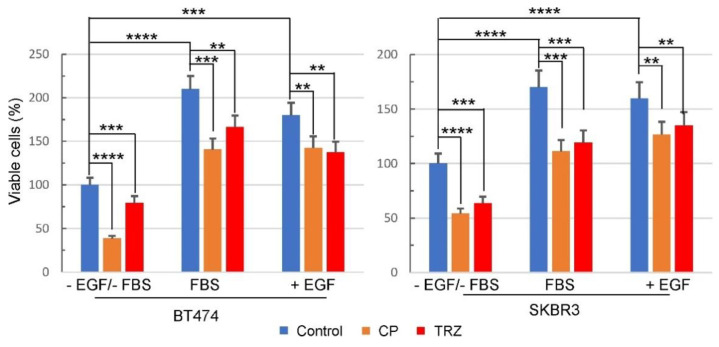
The effects of trastuzumab (TRZ) on the viability of BT474 and SKBR3 breast cancer cells in absence and presence of 10% FBS, and EGF. Cells were treated with 10 µg/mL concentration of trastuzumab for 72 h. Cells treated with normal human IgG were used as negative control and cells treated with CP–714724 (CP) at 1 µM concentration were used as positive control. Each value is the average of at least three experiments and the error bar is standard error. **: *p* < 0.01, ***: *p* < 0.001, ****: *p* < 0.0001.

We first determined if trastuzumab inhibits the proliferation of BT474 and SKBR3 cells. For this purpose, the MTT cell viability kit was used to assess cell proliferation. The cells were treated with trastuzumab for 72 h under three different conditions including: (1) DMEM only, without adding any supplements, (2) DMEM supplemented with 10% FBS, and (3) DMEM supplemented with EGF. It is worth mentioning that even though culturing the cells in the medium without FBS slowed the cell growth, it did not have adverse effects on the health of the cells. Our results showed that trastuzumab inhibited cell proliferation in the absence and the presence of 10% FBS and EGF in both BT474 and SKBR3 cells. As a positive control, we treated the cells with HER2-specific small molecule tyrosine kinase inhibitor, CP–724714, which inhibited the proliferation of BT474 and SKBR3 cells under all conditions (Figure 1).

**Figure 2:**
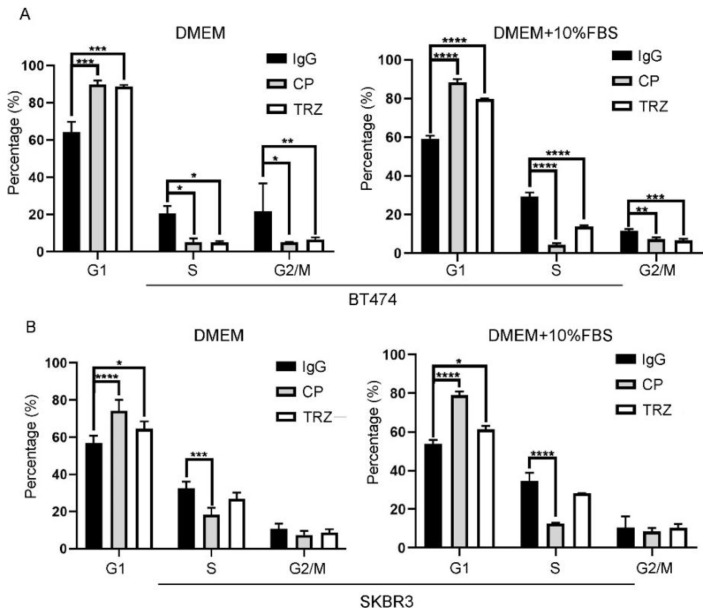
The effects of trastuzumab (TRZ) on the cell cycle progression of BT474 (**A**) and SKBR3 (**B**) cells in presence and absence of 10% FBS. Cells were treated with 10 µg/mL concentration of trastuzumab for 72 h. Cells treated with normal human IgG were used as negative control and cells treated with CP-714724 (CP) at 1 µM concentration were used as positive control. Each value is the average of at least three experiments and the error bar is standard error. *: *p* < 0.05, **: *p* < 0.01, ***: *p* < 0.001, ****: *p* < 0.0001.

Studies have revealed that trastuzumab inhibits the proliferation of HER2-positive breast cancer cells, likely through the inhibition of cell cycle progression [67,68,69,79,80,81]. To assess if this is the case for our observed inhibition in Figure 1, we examined if trastuzumab inhibits the cell cycle progression in BT474 and SKBR3 cells by flow cytometry. We treated the cells with trastuzumab at 10 µg/mL concentration in the presence and absence of 10% FBS for 72 h. We showed that trastuzumab treatment significantly increased the population of cells in G1 phase under all conditions in both BT474 and SKBR3 cells (Figure 2). As a positive control, CP-724714 also increased cell population in G1 phase similarly to trastuzumab (Figure 2). These data suggest that trastuzumab blocks cell proliferation by arresting cells in G1 phase.

### 3.2. The Effect of Trastuzumab on EGF-Induced Formation of EGFR–HER2 Heterodimers 

**Figure 3:**
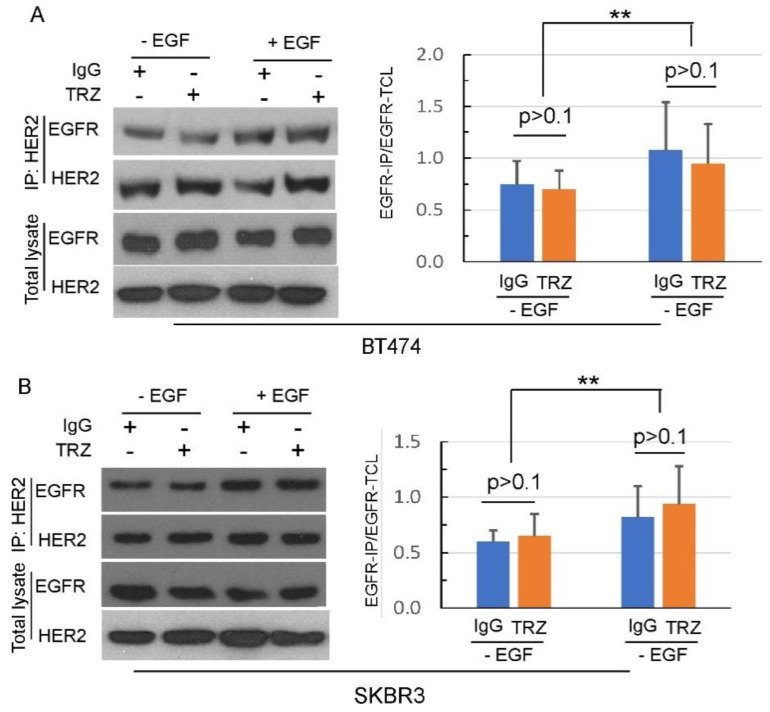
The effect of trastuzumab on HER2 heterodimer formation. The effect of trastuzumab at concentration of 10 µg/mL on HER2–EGFR and HER2–HER3 heterodimers in BT474 (**A**) and SKBR3 (**B**) breast cancer cells for 1 h. HER2–EGFR and HER2–HER3 heterodimer formation was assessed in the presence and absence of EGF and HRGα at 50 ng/mL, respectively. The expression of HER receptors in both IP and total cell lysate (TCL) samples was revealed by immunoblotting. HER2 receptors were precipitated using HER2-specific antibody as primary antibody, followed by immunoblotting with the indicated antibodies. Cells treated with normal human IgG were used as negative control. **: *p* < 0.01.

We next examined the effects of trastuzumab on EGF-induced formation of EGFR–HER2 heterodimers by co-immunoprecipitation (co-IP) assay in BT474 and SKBR3 cells. For this purpose, HER2 antibody was used as a primary antibody to precipitate HER2 receptors, and heterodimer formation was identified by immunoblotting of EGFR. We showed that HER2 formed a heterodimer with EGFR, with or without EGF stimulation. Quantification of the data showed that addition of EGF stimulated the dimerization between EGFR and HER2. Interestingly, trastuzumab did not block the heterodimerization (*p* > 0.1) between HER2 and EGFR with or without EGF stimulation in both BT474 and SKBR3 cells (Figure 3).

### 3.3. The Effect of Trastuzumab on the Phosphorylation of EGFR and HER2

**Figure 4:**
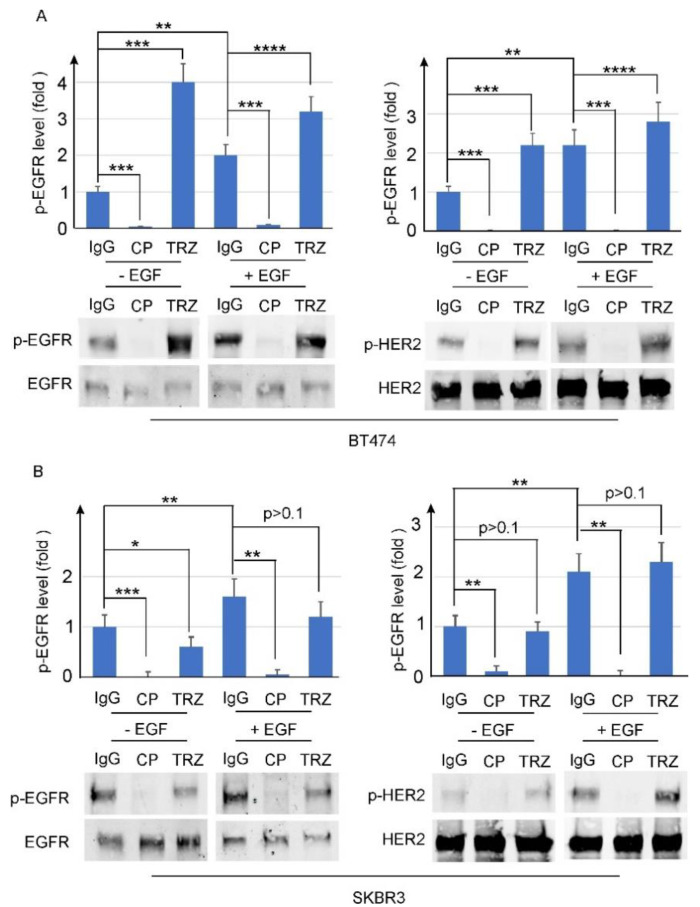
The effects of trastuzumab on the phosphorylation of HER receptors in BT474 (**A**) and SKBR3 (**B**) cells. The cells treated with trastuzumab at concentration of 10 µg/mL in the presence and absence of EGF at 50 ng/mL concentration. The phosphorylation of HER receptors was revealed by immunoblotting. Cells treated with normal human IgG were used as negative control and cells treated with CP–714724 (CP) at 1 µM concentration were used as positive control. Each value is the average of at least three experiments and the error bar is standard error. *: *p* < 0.05, **: *p* < 0.01, ***: *p* < 0.001, ****: *p* < 0.0001.

To determine the effect of trastuzumab on the phosphorylation of EGFR and HER2, we treated the BT474 and SKBR3 cells with trastuzumab (10 µg/mL) in the presence or absence of EGF for 1 h. The cells were also treated with IgG (10 µg/mL) as the negative control and CP-714724 (1 µM) as the positive control (Figure 4). We showed that EGF stimulated EGFR and HER2 phosphorylation in both cell lines, as expected. Interestingly, trastuzumab did not inhibit, but actually stimulated EGF-induced phosphorylation of EGFR and HER2 in BT474 cells. For SKBR3 cells, EGF did not inhibit or stimulate EGFR and HER2 phosphorylation (Figure 4).

**Figure 5:**
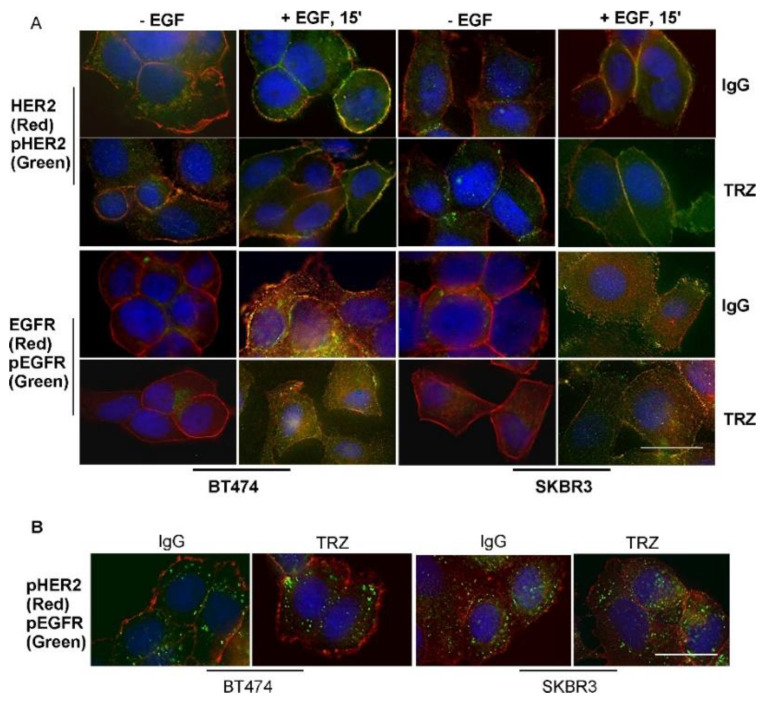
The effects of trastuzumab on EGF-induced phosphorylation of EGFR and HER2 in BT474 and SKBR3 cells. Cells were incubated with TRZ (10 µg/mL) or human IgG for 1 h and then stimulated with EGF (50 ng/mL). (**A**) Following EGF stimulation for 15 min, the phosphorylation of EGFR and HER2 (green) was determined by antibody to phosphorylated EGFR (pEGFR, pY1086) and pHER2 (pY1139), respectively. The localization of EGFR and HER2 (red) was determined by antibodies to by total EGFR and HER2, respectively. Yellow indicates the co–localization. the size bar = 20 µm. (**B**) Localization of pEGFR (pY1086) and pHER2 (pY1139) following EGF stimulation for 30 min. the size bar = 20 µm.

We further examined the effect of trastuzumab on the phosphorylation of EGFR and HER2 by immunofluorescence. Both BT474 and SKBR3 cells were treated with IgG (control) or trastuzumab for 1 h, and then stimulated with EGF. As shown in Figure 5A, in the absence of EGF, EGFR was slightly phosphorylated, but HER2 maintained a higher level of phosphorylation. GF stimulated strong phosphorylation of both EGFR and HER2. Treatment with trastuzumab did not show any observable inhibitory effects on the phosphorylation of EGFR and HER2. Following EGF stimulation for 30 min, phosphorylated EGFR was mostly internalized to the endosomes, but phosphorylated HER2 mostly stayed in the plasma membrane (Figure 5B).

**Figure 6:**
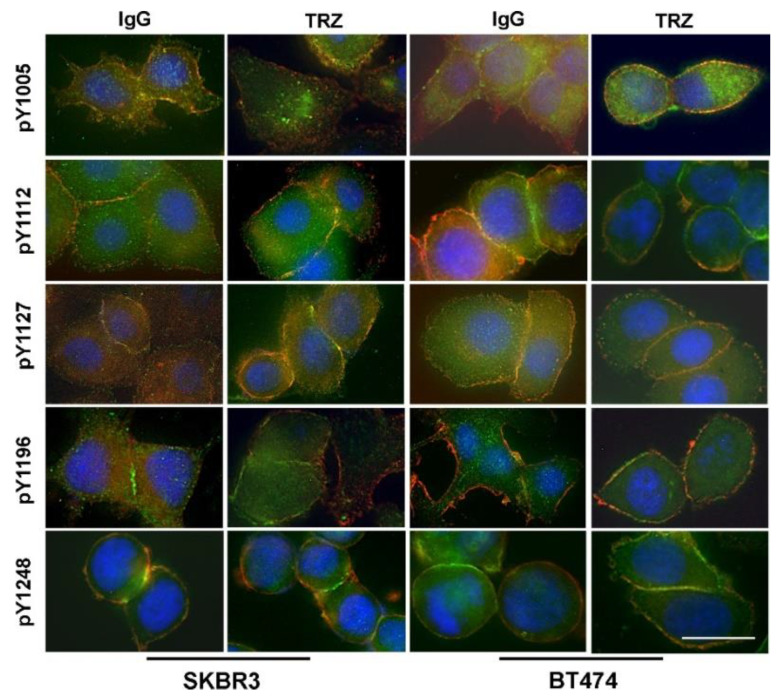
The effects of trastuzumab on EGF-induced phosphorylation of HER2 at various pY sites including pY1005, pY1112, pY1127, pY1196, and pY1248 in BT474 and SKBR3 cells. Cells were incubated with TRZ (10 µg/mL) or human IgG for 1 h and then stimulated with EGF (50 ng/mL) for 15 min. The phosphorylation of various HER2 pY sites (green) were determined by specific antibodies followed by FITC conjugated secondary antibody, and total HER2 (red) was revealed by anti-HER2 antibody followed by TRITC conjugated secondary antibody. Yellow indicates the co-localization. the size bar = 20 µm.

It has been shown that HER2 contains multiple phosphotyrosine (pY) residues that may be differentially regulated [51,58]. In the above experiments, we have been using antibody against HER2 pY1139 to determine the phosphorylation status of HER2. Here, we further examined the phosphorylation status of other HER2 pY residues including pY1005, pY1112, pY1127, pY1196, and pY1248 (Figure 6). We showed that EGF stimulated the phosphorylation of all the HER2 pY residues and trastuzumab did not have observable effects on the phosphorylation of these HER2 pY residues.

Together, our data indicated that EGF induced HER2 phosphorylation in both BT474 and SKBR3 cells. Trastuzumab did not inhibit HER2 phosphorylation at all major pY residues, with or without EGF stimulation. 

### 3.4. The Effect of Trastuzumab on HER2-Mediated Downstream Signaling Pathways

**Figure 7:**
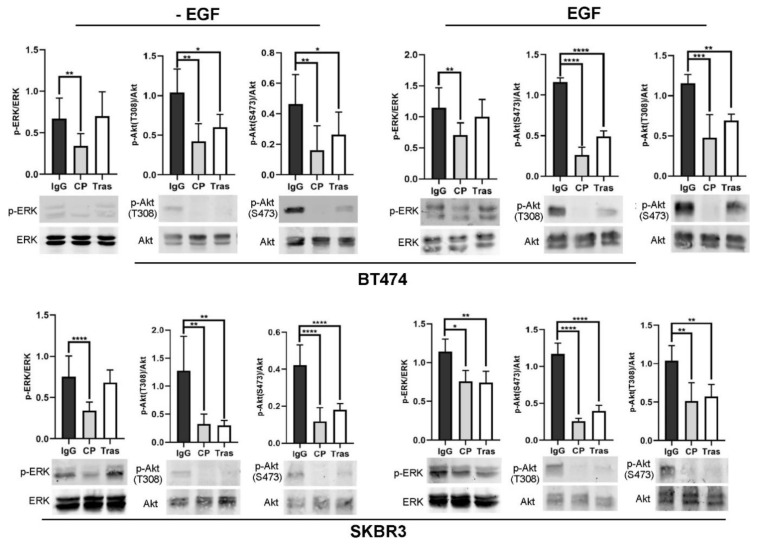
The effects of trastuzumab on HER2-mediated downstream signaling pathway in BT474 and SKBR3 cells. The cells were treated with trastuzumab at concentration of 10 µg/mL in the presence and absence of EGF (50 ng/mL). The phosphorylation of Akt at threonine (T) 308 and serine (S) 473 phosphorylation sites as well as Erk phosphorylation were revealed by immunoblotting. Cells treated with normal human IgG were used as negative control and cells treated with CP–714724 (CP) at 1 µM were used as positive control. Each value is the average of at least three experiments and the error bar is standard error. *: *p* < 0.05, **: *p* < 0.01, ***: *p* < 0.001, ****: *p* < 0.0001.

We next examined if trastuzumab exerted any inhibitory effects on HER2-mediated downstream signaling. The AKT pathway and ERK pathway are the two most studied and important signaling pathways downstream; EGFR and HER2 and were examined in this study. Both BT474 and SKBR3 cells were treated with trastuzumab (10 µg/mL) for 1 h and then stimulated with EGF (50 ng/mL) for 30 min. Similarly, treatment with IgG (10 µg/mL) and CP-714724 (1 µM) were used as negative control and positive control, respectively. The activation of AKT and ERK were determined by examining their phosphorylation status through immunoblotting. It is well established that the full activation of AKT requires the phosphorylation of both T308 and S473. We examined the effects of trastuzumab on the AKT T308 and S473 phosphorylation with specific antibodies, and showed that trastuzumab inhibited the AKT phosphorylation at both T308 and S473 phosphorylation sites, with or without EGF stimulation in both BT474 and SKBR3 cells (Figure 7). However, the effects of trastuzumab on the phosphorylation of ERK are weaker. Trastuzumab did not inhibit the basal ERK phosphorylation in both BT474 and SKBR3 cells. While it inhibited EGF-induced phosphorylation of ERK in SKBR3 cells, trastuzumab only slightly inhibited EGF-induced ERK phosphorylation in BT474 cells, which is statistically insignificant (Figure 7). Together, these results indicated that trastuzumab strongly inhibited EGF-induced Akt c.

### 3.5. The Effect of Trastuzumab on Lipid Raft Localization and Function of EGFR–HER2 Heterodimer

The above research revealed interesting effects of trastuzumab. In HER2-positive BT474 and SKBR3 cells, trastuzumab did not inhibit the dimerization of EGFR–HER2 receptors, and the phosphorylation of EGFR and HER2. However, trastuzumab strongly inhibited EGF-induced phosphorylation of AKT. Moreover, trastuzumab blocked proliferation of both BT474 and SKBR3 cells by arresting the cells in G1 phase. These results suggest that trastuzumab inhibits EGF-induced activation of AKT and cell cycle progression/proliferation by a mechanism other than the dimerization and phosphorylation of EGFR and HER2. 

Previous studies have shown that lipid raft localization of HER receptors, including EGFR and HER2, plays a critical role in the activation of downstream signaling pathways [22,82,83,84,85,86,87]. In addition, trastuzumab binds to the juxta-membrane region of HER2 [88], which likely affects the movement of the HER2 transmembrane domain within the plasma membrane. Indeed, it has been shown that trastuzumab can affect HER2 receptors’ localization and arrangement in the membrane of breast cancer cells [89,90]. Therefore, we proposed that trastuzumab inhibits the lipid raft localization of EGFR–HER2 heterodimer, which leads to the inhibition of Akt activation. 

**Figure 8:**
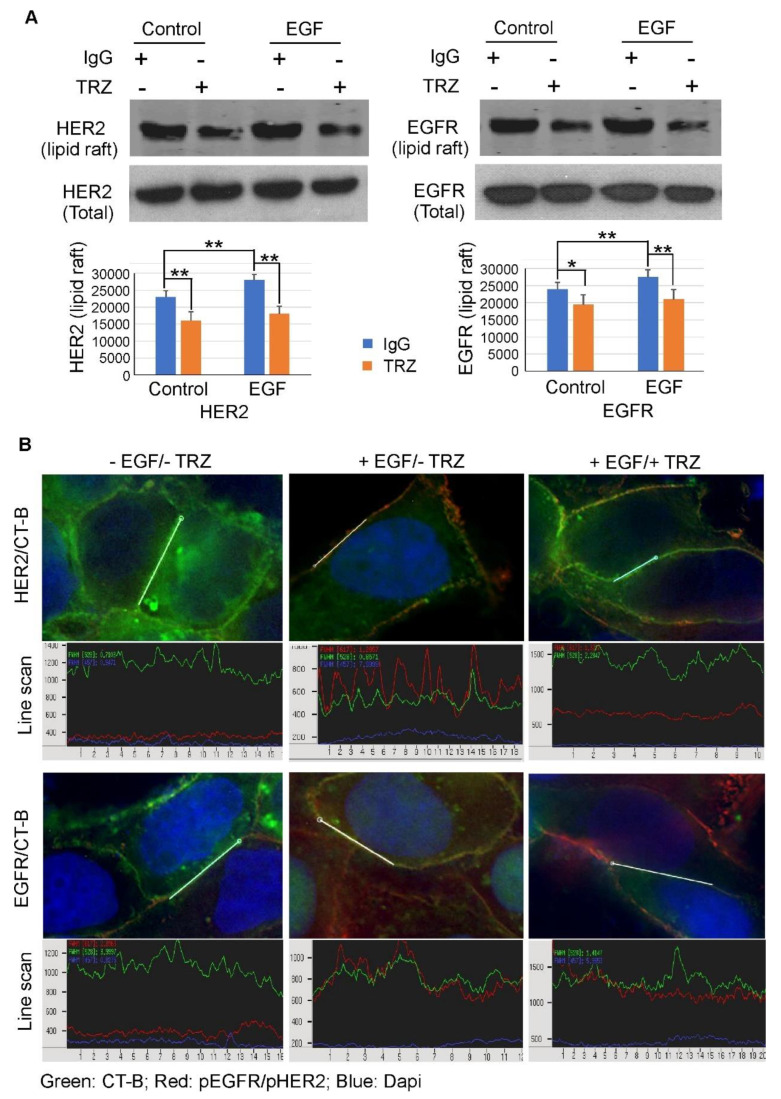
The effect of trastuzumab on EGF-induced lipid raft localization of EGFR–HER2 heterodimer in BT474 cells. (**A**) EGFR and HER2 level in isolated lipid rafts. The cells were incubated with trastuzumab or normal human IgG (10 µg/mL) for 1 h and then stimulated with EGF (50 ng/mL) for 30 min. Proteins associated with lipid rafts were isolated by using Bio-Rad ReadyPrep™ Protein Extraction Kit (Signal). The level of HER2 and EGFR receptors in isolated lipid rafts were examined by immunoblotting. Each value is the average of three experiments and the error bar is the standard error. *: *p* < 0.05, **: *p* < 0.01. (**B**) Co-localization (yellow) of pEGFR (red) and pHER2 (red) with Alex Fluor 488 conjugated CT-B (green). Cells were incubated with CB-T (10 µg/mL) with or without trastuzumab (10 µg/mL) for 1 h and then stimulated with EGF for 30 min. The localization of EGFR or HER2 was revealed by TRITC conjugated secondary antibody following the incubation with the primary antibody. Nucleus was counter stained with Dapi.

To test this hypothesis, we first investigated the possible effect of trastuzumab on HER2 and EGFR localization to the lipid raft microdomains of the cell membrane. We treated the cells with trastuzumab (10 µg/mL) with or without EGF for 1 h, and isolated the proteins associated with lipid rafts using Bio-Rad ReadyPrep™ Protein Extraction Kit (Signal). The cells treated with IgG were used as the negative control. The level of HER2 and EGFR in isolated lipid rafts were examined by immunoblotting. We showed that EGF stimulated the translocation of EGFR and HER2 to the lipid raft. Moreover, the localization of HER2 and EGFR to the lipid rafts was decreased after the treatment of cells with trastuzumab in both BT474 and SKBR3 cells (Figure 8A).

We further studied the effects of trastuzumab on the lipid raft localization of EGFR and HER2 by indirect immunofluorescence. Alexa Fluor 488 conjugated cholera toxin subunit (CT-B), which binds to the raft constituent ganglioside GM1, was used as the marker for lipid rafts. The co-localization of EGFR/HER2 and CTB with or without EGF stimulation were examined by fluorescence microscopy. As shown in Figure 8B, the peaks of green indicated the location of lipid rafts along the PM (line scan). In the absence of EGF stimulation, both EGFR and HER2 (red) distributed smoothly along the PM, not concentrated in the lipid rafts. Following EGF stimulation for 30 min, the intensity peak of HER2 and EGFR (red) overlapped with the peak of CT-B (green) as shown in line scan, indicating the translocation of EGFR and HER2 to lipid rafts. However, pretreatment with trastuzumab blocked the lipid translocation of both HER2 and EGFR. Both HER2 and EGFR showed smooth distribution along the PM, and were not co-peaked with CT-B as shown in the line scan. 

Together, our data indicated that trastuzumab strongly blocked EGF-induced translocation of pHER2 and pEGFR to the lipid rafts (Figure 8).

**Figure 9:**
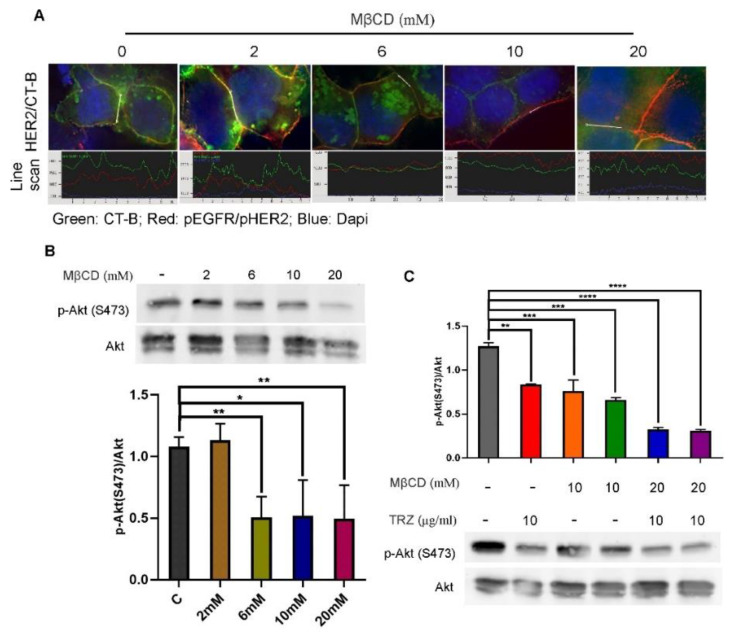
Disruption of lipid raft by MβCD and the effects on EGF-induced Akt phosphorylation. BT474 cells were treated with MβCD at various concentrations for 1 h to disrupt lipid raft. For the experiments with trastuzumab treatment, cells were incubated with trastuzumab and MβCD together for 1 h. Cells were then stimulated with EGF for 30 min. (**A**) CT-B stain (green) to show the disruption of lipid raft by MβCD. Cells were also stained with pHER2 (red). Line scan showed the intensities of CT-B and pHER2. (**B**) Dose-dependent effects of MβCD on Akt phosphorylation in BT474 breast cancer cells. Cells were treated with MβCD at indicated concentration for 4 h. The phosphorylation of Akt was then examined by immunoblotting. (**C**) Synergistic effects of trastuzumab and MβCD on Akt phosphorylation. Cells were treated with MβCD alone at 10 and 20 mM concentrations, or in combination with trastuzumab at 10 μg/mL. The phosphorylation of Akt was then examined by immunoblotting. *: *p* < 0.05, **: *p* < 0.01, ***: *p* < 0.001, ****: *p* < 0.0001.

We next determined if the disruption of the lipid raft would block EGF-induced Akt phosphorylation. It has been demonstrated that methyl-β-cyclodextrin (MβCD) can disrupt the cell membrane lipid raft through depletion of cell membrane cholesterol [13]. We treated the cells with different concentrations of MβCD to disrupt the lipid raft, and examined the effects on AKT phosphorylation. We first examined if the lipid raft is disrupted by MβCD, by examining the binding of CT-B to the membrane (Figure 9A). As shown in Figure 9A, with the increase in MβCD concentration, the binding of CTB to the membrane decreased. CTB was hardly visible when MβCD concentration was at 20 µM. The intensity data indicated in the line scan showed that CT-B intensity (green) dropped from approximately 2000 (no MβCD) to 700 (20 mM MβCD), indicating that the lipid raft is significantly disrupted by 20 µM MβCD (Figure 9A). The intensity of HER2 was barely changed under all treatment conditions (Figure 9A).

We then examined the effects on EGF-induced Akt phosphorylation. We showed that MβCD suppresses AKT phosphorylation in BT474 cells in a dose-dependent manner (Figure 9B), which is consistent with the dose-dependent disruption of lipid rafts (Figure 9A). Moreover, the inhibitory effects of MβCD on AKT phosphorylation are comparable to the inhibitory effects of trastuzumab, and the combination of MβCD and trastuzumab produced stronger inhibition (Figure 9C). 

Together, our data suggest that Trastuzumab likely inhibits HER2-mediated cell signaling by blocking its translocation to the lipid raft, which is critical for the activation of downstream Akt signaling. 

## 4. Discussion

To improve the efficacy of trastuzumab, it is essential to understand its mechanism of action. While many studies have been performed to reveal how trastuzumab affects the intracellular signaling of breast cancer cells, the data are controversial and fail to provide a clear picture. One of the significant issues that makes it difficult to determine the precise mechanism of trastuzumab action is the formation of various HER receptor dimers in HER2-positive breast cancer cells. A significant portion of HER2-positive breast cancer cells co-express EGFR and HER3 [91], and HER2-mediated cell signaling, and cell function is closely related to and impacted by other HER receptors [1]. It is not clear how the formation of various HER receptors’ homo- and heterodimers impact the function of trastuzumab in HER2-mediated cell signaling, and how this is related to the efficacy of trastuzumab in treating breast cancer. So far, the studies have been focused on the role of HER2–HER3 heterodimers, as HER3 has multiple PI3K binding sites [9,10,92]. However, it is well established that EGFR does not only activate the Ras/Erk pathway, but also strongly activates the PI3K/Akt pathway through multiple mechanisms [8,93,94]. Therefore, we focused our study on the role of trastuzumab on the cell signaling and cell proliferation mediated by EGFR–HER2 heterodimers in BT474 and SRBR3 cells.

EGF stimulates the formation of both EGFR homodimer and EGFR–HER2 heterodimer. Trastuzumab only binds to HER2, not EGFR. Therefore, any effects of trastuzumab on EGF-induced activation of EGFR, HER2, and downstream signaling proteins, as well as cell proliferation, are through its effects on EGFR–HER2 heterodimers. 

We showed that trastuzumab inhibits EGF- and FBS-induced cell proliferation in both BT474 and SKBR3 cells (Figure 1). This observation is consistent with previous publications [79,95]. Moreover, we showed that trastuzumab blocks cell cycle progression by arresting cells in G1 phase (Figure 2). Although several studies have shown that trastuzumab arrests the cell cycle at G1 phase [67,69,81,96], no study has assessed the effect of trastuzumab on cell cycle progression in the presence of EGF. 

We further showed that trastuzumab does not block EGF-induced EGFR–HER2 heterodimerization (Figure 3). Differently from pertuzumab, which binds to the HER2 dimerization loop and inhibits HER2 dimerization, trastuzumab binds to the HER2 domain IV, and should not interfere with HER2 dimerization. Indeed, we previously showed that trastuzumab does not block HER2 homodimerization [51]. Other early studies also showed that trastuzumab does not block the heterodimerization of HER2 with EGFR and HER3 [79,95]. Our finding further indicates that trastuzumab does not interfere with HER2 dimerization. 

We next studied the effects of trastuzumab on EGF-induced phosphorylation of EGFR and HER2. We showed that trastuzumab does not block EGF-induced phosphorylation of EGFR and HER2 in SKBR3 cells, and actually increases the phosphorylation of EGFR and HER2 in BT474 cells (Figure 4 and Figure 5). When individual tyrosine residues were examined by immunofluorescence, we also showed that trastuzumab does not block the phosphorylation of any of the known phosphotyrosine residues in the HER2 C-terminus including pY1005, pY1112, pY1127, pY1139, pY1196, and pY1248 (Figure 6). This is not surprising, as we have shown that trastuzumab does not interfere with EGF-induced heterodimerization of EGFR and HER2. Our data are also consistent with previous publications [40,41,42,58,64].

We also studied the effects of trastuzumab on the activation (phosphorylation) of signaling proteins downstream, EGFR and HER2. Previous studies regarding trastuzumab action and resistance in breast cancer have been focused on its role in the Akt pathway, in the context of HRG-induced HER2–HER3 heterodimers [8,60,67,68,69,70,71,72,73,74,75,76,77]. Very little is known about the effects of trastuzumab on the signaling pathway mediated by EGF-induced EGFR–HER2 heterodimers. We showed that trastuzumab strongly inhibits EGF-induced phosphorylation of Akt at both T308 and S473, and slightly inhibits EGF-induced phosphorylation of Erk (Figure 7). This finding is consistent with our observation that trastuzumab inhibits cell proliferation and cell cycle progression in BT474 and SKBR3 cells (Figure 1 and Figure 2). 

However. this finding is quite surprising giving our data regarding the effects of trastuzumab on EGF-induced EGFR and HER2 heterodimerization and phosphorylation (Figure 3, Figure 4, Figure 5 and Figure 6). How is it that trastuzumab, which specifically binds to HER2, did not block HER2–EGFR dimerization, did not inhibit EGFR and HER2 phosphorylation, yet inhibited Akt phosphorylation and the growth of BT474 and SKBR3 in response to EGF? 

Indeed, this hypothesis is supported by our data (Figure 8 and Figure 9). We first showed that EGF stimulates the translocation of HER2 and EGFR to the lipid raft, which suggests that lipid raft localization of EGFR/HER2 is involved in their signaling (Figure 8). We further showed that treatment with trastuzumab blocked this EGF-induced translocation of both EGFR and HER2 (Figure 8). This suggests that trastuzumab exerts its effects through EGFR–HER2 heterodimer because HER2 only responds to EGF when it forms a heterodimer with EGFR, and EGFR only responds to trastuzumab when it forms a heterodimer with HER2. These data also indicate that trastuzumab can interfere with EGF-induced cell signaling by blocking the lipid raft translocation of EGFR–HER2 heterodimers. 

We finally showed that disrupting the lipid raft with MβCD blocks the localization of HER2 to the lipid raft and blocks Akt phosphorylation in response to EGF (Figure 9). MβCD-induced disruption of the lipid raft and suppression of AKT phosphorylation are parallel in a dose-dependent manner (Figure 9A,B). Moreover, the effects of MβCD on AKT phosphorylation are comparable to the effects of trastuzumab, and a combination of MβCD and trastuzumab produced stronger inhibition (Figure 9C). Together, we showed that trastuzumab functions through a novel mechanism: blocking the translocation of EGFR–HER2 heterodimers to the lipid raft that is required for the activation of Akt by the EGFR–HER2 heterodimer.

## 5. Conclusions

Trastuzumab does not inhibit EGF-induced dimerization of EGFR and HER2 and does not inhibit EGF-induced phosphorylation of EGFR and HER2, in both BT474 and SKBR3 cells. However, trastuzumab inhibits EGF-induced Akt phosphorylation and cell proliferation by blocking EGF-induced translocation of EGFR–HER2 heterodimers to lipid rafts.

## Data Availability

Not applicable.
